# Supplementary material for: Research on the impact of enterprise mergers and acquisitions on technological innovation: An empirical analysis based on listed Chinese enterprises
Source: PLoS One. 2024 Nov 27;19(11):e0309569. doi: 10.1371/journal.pone.0309569 (PMC11602071; doi:10.1371/journal.pone.0309569)
Supplement: S1 File — (ZIP) [file pone.0309569.s001.zip › S1 File/Appendix 1.docx]

**Appendix 1.**

| **Abbreviation words** | **Meaning** |
| --- | --- |
| CSMAR | China Stock Market & Accounting Research |
| M&A | Mergers and acquisitions |
| R&D | Research and development |
| $Firmnum$ | Number of companies in the industry |
| $Sizeind$ | Industry average company size |
| $HHI$ | Degree of competition in the industry, calculated using the industry Herfindahl index |
| $DID$ | Difference-in-Differences |
| $Treat_{it+p}$ | Dummy variables. Firm $i$ has a merger in year $t$relative to year p, which takes the value of 1, and other years take the value of 0 |
| $Post$ | Dummy variables. The year in which the firm's M&A occurs is taken as the base year, with the current period taken as 1 and all other years taken as 0 |
| $did_{it+p}$ | Dummy variables. The product of the relative year $p$ in which firm $i$ had a merger in year $t$ and the base year of the firm's mergers and acquisitions |
| $Treat\_t$ | Dummy variables. Setting up to advance the actual occurrence of mergers and acquisitions by$t$ years, where$t$ is taken to be 1, 2, 3, or 4 |
| $did\_t$ | Dummy variables. The product of the year $t$ in advance of the actual merger or acquisition and the base year of the merger or acquisition, where $t$ is taken to be 1, 2, 3 or 4 |
| $lnnum$ | Annual cumulative values of articles, conferences, newspapers, etc., searched on China Knowledge Network with the keyword of “mergers and acquisitions”, plus 1 to take the logarithm |
| $TFP\_LP$ | Total factor productivity by Levinsohn-Petrin methodology |
| $Digit$ | Digital Transformation. Using enterprise company annual report artificial intelligence technology, big data technology, cloud computing technology, blockchain technology, digital technology application keywords word frequency plus 1 to take the natural logarithm of measurement |
| $ias$ | Intangible assets. Ratio of intangible assets to total assets plus 1 taken as natural logarithm |
| $lerner$ | Market power. (Operating Revenues - Costs)/Operating Revenues |
