# Supplementary material for: Research on the impact of enterprise mergers and acquisitions on technological innovation: An empirical analysis based on listed Chinese enterprises
Source: PLoS One. 2024 Nov 27;19(11):e0309569. doi: 10.1371/journal.pone.0309569 (PMC11602071; doi:10.1371/journal.pone.0309569)
Supplement: Attachment — Submitted filename: Reviewer.docx [file pone.0309569.s003.docx]

In the manuscript titled *Research on the Mechanism and Path of Digital Transformation Driving Technological Innovation: Based on the Perspective of Enterprise Mergers and Acquisitions*, Authors use panel data from A-share listed companies in Shanghai and Shenzhen from 2007 to 2020 to construct a theoretical framework for digital transformation driving technological innovation from the perspective of mergers and acquisitions. The research content of this study is rich. However, the lack of clarity of the research question is the major flaw of the study. Therefore, MAJOR revision has to be done before this manuscript could be accepted for publication in the *PLOS ONE*.

1. The logic is confusing and the research question is not clear. The title of the article is Research on the Mechanism and Path of Digital Transformation Driving Technological Innovation: Based on the Perspective of Enterprise Mergers and Acquisitions. From the perspective of the research content, the paper introduces the impact and path of mergers and acquisitions on technological innovation. However, it does not address the question of whether digital transformation can drive technological innovation and its internal mechanism.

2. In essence that paper is too long and boring with a large use of mathematics that is often used without clarity and sufficient motivations.

3. In the second part, it would be beneficial to supplement the literature on corporate mergers and acquisitions.

4. In part 4, there is a lack of information on indicators and methods for measuring digital transformation.

5. Part introduction should be shortened especially the political one. It is not scientifically sound.

6. Discussion is rather poor. What is interesting is to discuss your results with already published as well as their importance for the whole scientific community. What is new that you add to the existing body of knowledge, what are strengths and limitations of the study.

7. The policy recommendations needs to be further improved, and it is suggested that the author further expand the policy based on the core research results.
