# Supplementary material for: Research on the impact of enterprise mergers and acquisitions on technological innovation: An empirical analysis based on listed Chinese enterprises
Source: PLoS One. 2024 Nov 27;19(11):e0309569. doi: 10.1371/journal.pone.0309569 (PMC11602071; doi:10.1371/journal.pone.0309569)
Supplement: Attachment — Submitted filename: Response to Reviewers.docx [file pone.0309569.s004.docx]

**Respond to the editor’ comments**

Dear Editor:

Thank you for your letter and the constructive comments on our manuscript, “Research on the Mechanism and Path of Digital Transformation Driving Technological Innovation: Based on the Perspective of Enterprise Mergers and Acquisitions” (PONE-D-24-14261), in your busy schedule. All of us authors have carefully read the comments that you have given us, and have discussed and revised each of these issues. The following is my list of revisions. In addition, we have resubmitted a new manuscript in the revised state, with the revisions highlighted in red. If there are any incorrect answers or questions in the manuscript, please do not hesitate to let us know.

Based on the comments of both reviewers and my own assessment, I am recommending a major revision decision. I urge you to review your introduction and abstract, and make it more structured, offering a clear perspective of your problem, gap, research question, method and contribution, all in a connected an dlogical order. I also highlight the necessity to translate the values in Tuan to dollar, to make it a more clear reference. Please, carefully assess the comments from reviewer # 1, and if possible, perfomr a professional English review.

Response:

Thank you for your constructive comments. We shortened the abstract and revised the abstract section following the logic of the problem statement, methodology, findings, and recommendations.

Then we combine the suggestions of the editors and reviewers to reorganize the introduction section, and in order to make the introduction section more organized, we modify it according to the logic of real-life scenarios, case enumeration, research questions and methods. For the research contribution, this article puts the part in the Discussion section.

We have converted all Yuan values in the article to dollar values in order to make it a clearer reference.

After carefully evaluating commenter #1's comments, we invited a native English-speaking scholar to help embellish our article. We hope that the revised manuscript will be acceptable to you.

**Respond to the Reviewer #1’ comments**

Dear reviewer.

Thank you for your decision and constructive comments on my manuscript, “Research on the Mechanism and Path of Digital Transformation Driving Technological Innovation: Based on the Perspective of Enterprise Mergers and Acquisitions” (PONE-D-24-14261). These comments are valuable for the revision and improvement of our paper, and they are also important guidance for our research. We have carefully considered these comments and revised our thesis for your approval. In the revised original manuscript, all changes are marked in red to facilitate your review.

Revision notes, point-to-point, are given as follows:

1. The Abstract is too long. Need to shorten the abstract.

Response:

Thank you for your suggestion. We are gradually revising and shortening the abstract section along the lines of problem statement, methodology, findings and recommendations.

The revised abstract is below:

As an important means for enterprises to acquire technological resources, the impact of mergers and acquisitions on technological innovation and the underlying mechanism deserve in-depth study. Using the merger and acquisition data from 2007 to 2020 of Chinese A-share listed companies in Shanghai and Shenzhen, the causal effect and influence mechanism between mergers and acquisitions and technological innovation are identified and tested using the Difference-in-Differences method. The study finds that mergers and acquisitions have a long-term, sustained, technological innovation-enhancing effect on firms. Mechanism tests show that mergers and acquisitions can promote the technological innovation of enterprises by improving production efficiency, enriching digital knowledge, and enhancing market power. A heterogeneity analysis shows that the technological innovation enhancement effect of mergers and acquisitions is more significant when the mergers and acquisitions meet domestic merger and acquisition requirements, when there is a small size of transactions, and when the merger and acquisition enterprises involved are non-state-owned. It is suggested that enterprises and the government should take multiple measures, while paying attention to the impact of heterogeneity, to give full play to the positive effects of mergers and acquisitions on technological innovation.

2. Rearrange abstract: Include problem statements, objectives, methods, findings, and recommendations step by step.

Response:

Thank you very much for your suggestions. We have reorganized the summary to gradually follow the problem statement, methodology, findings and recommendations. The details are as follows:

Problem statement: As an important means for enterprises to acquire technological resources, the impact of mergers and acquisitions on technological innovation and the underlying mechanism deserve in-depth study.

Methodology: Using the merger and acquisition data from 2007 to 2020 of Chinese A-share listed companies in Shanghai and Shenzhen, the causal effect and influence mechanism between mergers and acquisitions and technological innovation are identified and tested using the Difference-in-Differences method.

Findings: The study finds that mergers and acquisitions have a long-term, sustained, technological innovation-enhancing effect on firms. Mechanism tests show that mergers and acquisitions can promote the technological innovation of enterprises by improving production efficiency, enriching digital knowledge, and enhancing market power. A heterogeneity analysis shows that the technological innovation enhancement effect of mergers and acquisitions is more significant when the mergers and acquisitions meet domestic merger and acquisition requirements, when there is a small size of transactions, and when the merger and acquisition enterprises involved are non-state-owned.

Recommendations: It is suggested that enterprises and the government should take multiple measures, while paying attention to the impact of heterogeneity, to give full play to the positive effects of mergers and acquisitions on technological innovation.

3. The very first paragraph of introduction section is not clear as mentioned; the 2023 work report of Government of state council further highlights what? and how will you relate it with your current study. I think it is a poor start of introduction chapter.

Response:

Thank you very much for your suggestion. We have revised the first paragraph in the Introduction section based on your suggestion. The details are in the first paragraph of Part I, Introduction.

Revision of the contents of the introduction is as follows:

In 2010, China has become the largest country in manufacturing industry, but has long been trapped in the plight of serious duplication of industry construction, low industrial concentration, weak independent innovation ability, and weak market competitiveness. Under the pattern of latecomer competition, breaking through the technological blockade, solving the “neck” problem, and realizing subversive innovation is the necessary way for Chinese enterprises to catch up with the technology. However, due to the lack of technical talents and the long technology research and development cycle, it is relatively slow for enterprises to rely on independent innovation to improve their technological level (Sear,2018；Guo et al.,2018). According to the theory of open innovation, enterprises can rapidly acquire heterogeneous knowledge that is difficult to purchase in the factor market through mergers and acquisitions (Li et al.,2019), and utilize external digital resources such as talent, technology and knowledge acquired through mergers and acquisitions to improve the level of technological innovation of enterprises (Cefis et al.,2020). With the growing demand for technological innovation in China, enterprises need to absorb new technological knowledge through cross-industry integration to realize technological innovation faster in order to maintain competitive advantages and grasp new opportunities (Fang et al.,2022), thus a large number of enterprises have emerged to acquire external knowledge through mergers and acquisitions to enhance technological innovation capability.

4. Rearrange the structure of Introduction section

Response:

Thank you very much for your comments. We have revised the introduction section according to your suggestions. In order to make the introduction section more organized, we modified it according to the logic of realistic scenarios, case examples, research questions and methods. The details are in Part I, Introduction.

The contents of the revised introduction are as follows:

In 2010, China has become the largest country in manufacturing industry, but has long been trapped in the plight of serious duplication of industry construction, low industrial concentration, weak independent innovation ability, and weak market competitiveness. Under the pattern of latecomer competition, breaking through the technological blockade, solving the “neck” problem, and realizing subversive innovation is the necessary way for Chinese enterprises to catch up with the technology. However, due to the lack of technical talents and the long technology research and development cycle, it is relatively slow for enterprises to rely on independent innovation to improve their technological level (Sear,2018；Guo et al.,2018). According to the theory of open innovation, enterprises can rapidly acquire heterogeneous knowledge that is difficult to purchase in the factor market through mergers and acquisitions (Li et al.,2019), and utilize external digital resources such as talent, technology and knowledge acquired through mergers and acquisitions to improve the level of technological innovation of enterprises (Cefis et al.,2020). With the growing demand for technological innovation in China, enterprises need to absorb new technological knowledge through cross-industry integration to realize technological innovation faster in order to maintain competitive advantages and grasp new opportunities (Fang et al.,2022), thus a large number of enterprises have emerged to acquire external knowledge through mergers and acquisitions to enhance technological innovation capability.

According to CSMAR data, in terms of quantity and amount, China's merger and acquisition activities have increased over the past decade (Ren et al.,2017). In 2020, the number of China's merger and acquisition transactions reached a record high, with a total of 9,879 merger and acquisition events and a total merger and acquisition value of $409.969 billion, up 168% and 356%, respectively, compared with 2007. However, there are many cases of failure. For example, since 2015, Chengdu Tianxiang Environment Co., Ltd., China's leading environmental protection equipment company, has acquired the U.S. sludge treatment equipment company Centrisys Corporation, Germany's Belfinger Water Treatment (BWT), and Germany's “solid waste giant” Euromonitor Group, but the company went bankrupt and delisted in December 2020 due to a lack of breakthroughs in core technology and poor operations.

Based on the above, this study aims to answer the following questions: Can mergers and acquisitions promote the technological innovation of Chinese enterprises? If so, what is the mechanism? As there are both state-owned and non-state-owned enterprises in China, does the relationship between mergers and acquisitions and technological innovation reflect heterogeneity according to the characteristics of enterprises and types of mergers and acquisitions? To address these questions, this paper identifies and examines the causal effect and influence mechanism between mergers and acquisitions and technological innovation based on the merger and acquisition data of Chinese A-share listed firms in Shanghai and Shenzhen under the framework of the Difference-in-Differences method of empirical testing. This paper further examines the impact of heterogeneity, i.e., firm idiosyncrasies, according to the type of merger and acquisition transactions and the nature of firm ownership.

5. Provide a separate section of limitations of current work.

Response:

Thank you very much for your suggestion. We have added limitations and future research in Section 6, Discussion, of the manuscript.

The revised limitations and future research are as follows:

Nonetheless, this study has some limitations. First, this study focuses on data from listed companies in China and attempts to consider nonlisted companies in the subsequent period. Second, this study observes the acquiring firms in merger and acquisition transactions, and the traits of the acquired firms, as the other participants in merger and acquisition transactions, may also have an impact on technological innovation. Third, this study is based on data from Chinese listed companies in the Chinese context. There are significant differences in the merger and acquisition behaviors and external conditions of firms in different countries. Therefore, future research can be carried out in the following three ways. First, nonlisted companies can be used as the research object in the subsequent period to comprehensively understand the impact of mergers and acquisitions on technological innovation in Chinese enterprises. Second, the acquired enterprises can be used as the research object to further enrich the body of knowledge on the impact of mergers and acquisitions on technological innovation. Third, to make the results of the study more widely applicable, future enterprise samples should be obtained based on a larger number of countries to conduct empirical tests.

6. Add seperate table for abbreviations used in this research work.

Response:

Thank you very much for your suggestion. We have added a separate table to explain the meaning of abbreviated words in the manuscript. The details are in Appendix 1 of the manuscript.

The revised table is below:

**Appendix 1.**

| **Abbreviation words** | **Meaning** |
| --- | --- |
| CSMAR | China Stock Market & Accounting Research |
| M&A | Mergers and acquisitions |
| R&D | Research and development |
| $Firmnum$ | Number of companies in the industry |
| $Sizeind$ | Industry average company size |
| $HHI$ | Degree of competition in the industry, calculated using the industry Herfindahl index |
| $DID$ | Difference-in-Differences |
| $Treat_{it+p}$ | Dummy variables. Firm $i$ has a merger in year $t$relative to year p, which takes the value of 1, and other years take the value of 0 |
| $Post$ | Dummy variables. The year in which the firm's M&A occurs is taken as the base year, with the current period taken as 1 and all other years taken as 0 |
| $did_{it+p}$ | Dummy variables. The product of the relative year $p$ in which firm $i$ had a merger in year $t$ and the base year of the firm's mergers and acquisitions |
| $Treat\_t$ | Dummy variables. Setting up to advance the actual occurrence of mergers and acquisitions by$t$ years, where$t$ is taken to be 1, 2, 3, or 4 |
| $did\_t$ | Dummy variables. The product of the year $t$ in advance of the actual merger or acquisition and the base year of the merger or acquisition, where $t$ is taken to be 1, 2, 3 or 4 |
| $lnnum$ | Annual cumulative values of articles, conferences, newspapers, etc., searched on China Knowledge Network with the keyword of “mergers and acquisitions”, plus 1 to take the logarithm |
| $TFP\_LP$ | Total factor productivity by Levinsohn-Petrin methodology |
| $Digit$ | Digital Transformation. Using enterprise company annual report artificial intelligence technology, big data technology, cloud computing technology, blockchain technology, digital technology application keywords word frequency plus 1 to take the natural logarithm of measurement |
| $ias$ | Intangible assets. Ratio of intangible assets to total assets plus 1 taken as natural logarithm |
| $lerner$ | Market power. (Operating Revenues - Costs)/Operating Revenues |

7. English used in this work is too weak, found grammatically error which has greater impact on this current paper and also can degrade the value of this journal.

Response:

Thanks for the suggestion. We invited a native English-speaking scholar to help embellish our article. We hope the revised manuscript will be acceptable to you.

8. References are not in alphabetical order. Rearrange them.

Response:

Thank you very much for your comments. We have listed the references in alphabetical order, as specified at the references.

**We have done our best to improve the manuscript, and have made some red-flagged changes in revising the paper that do not affect the content or framework of the paper. We sincerely thank the editor and reviewers for their enthusiastic work and hope that the revisions will be recognized. Thank you again for your comments and suggestions.**

**Respond to the Reviewer #2’ comments**

Dear reviewer.

We would like to thank you very much for your professional comments on our manuscript "Research on the Mechanisms and Paths of Digital Transformation Driving Technological Innovation: Based on the Perspective of Enterprise Mergers and Acquisitions" (PONE-D-24-14261). These comments are valuable for the revision and improvement of our paper, and they are also important guidance for our research. Based on your suggestions, we have made extensive corrections to our previous manuscript, with corrections marked in red for your review.

The following is a point-by-point response to your comments.

1. The logic is confusing and the research question is not clear. The title of the article is Research on the Mechanism and Path of Digital Transformation Driving Technological Innovation: Based on the Perspective of Enterprise Mergers and Acquisitions. From the perspective of the research content, the paper introduces the impact and path of mergers and acquisitions on technological innovation. However, it does not address the question of whether digital transformation can drive technological innovation and its internal mechanism.

Response:

We are very grateful for your valuable comments. Regarding the problem of unclear research questions, we have reorganized and summarized the research questions and ideas of the manuscript to make it clear that this article mainly addresses the research question of the impact of enterprise mergers and acquisitions on technological innovation. At the same time, we have also revised the last paragraph of the introduction to address the issue and clarify the research problem of the article. Therefore, we modify the title of the article to "Research on the impact of enterprise mergers and acquisitions on technological innovation: An empirical analysis based on Chinese listed enterprises". The specific research framework is shown in Figure 1 below:

**Fig 1. Research framework.**

2. In essence that paper is too long and boring with a large use of mathematics that is often used without clarity and sufficient motivations.

Response:

Thank you for your suggestion, we have sorted out the problems and deleted and revised the content of the paper. The main research ideas and contents of this paper are as follows:

This study combined with the reality found that technological innovation is an important driving force for the development of national enterprises, and relying on the independent innovation of enterprises to enhance the level of technology is relatively slow, mergers and acquisitions, as an important way for enterprises to quickly access emerging knowledge and technology, has gradually become an important way for enterprises to realize technological innovation. Therefore, this paper researches and analyzes the relationship between corporate mergers and acquisitions and technological innovation.

First of all, this study respectively on enterprise mergers and acquisitions, technological innovation, enterprise mergers and acquisitions and technological innovation of the relationship between the literature collation summarization found that mergers and acquisitions on technological innovation impact and mechanism of the path of the research is quite limited, combined with the background of the times, case study analysis found that enterprise mergers and acquisitions on technological innovation of the impact of the mechanism of the path of the study has a certain significance of the reality, is worth the theoretical community and the practical community of the in-depth investigation.

At the same time, this study analyzes the mechanism and research hypotheses on the relationship between corporate mergers and acquisitions and technological innovation, and then empirically tests the research hypotheses, and then chooses different methods, such as parallel trend test, placebo test and endogeneity test, to conduct a robustness test on the core conclusions, and the test results show that the conclusions still hold.

In addition, in order to explore the mechanism path of M&A to promote technological innovation, this study discusses the three aspects of improving production efficiency, enriching digital knowledge, and enhancing market power, and constructs the corresponding model to empirically test the relationship between the variables, and the study shows that M&A can promote technological innovation through the three paths of improving production efficiency, enriching digital knowledge, and enhancing market power.

Then, in order to explore the heterogeneity of the impact of M&A on technological innovation, the study on the ownership of M&A enterprises, the type of M&A, the amount of M&A grouped research, the study found that, compared with state-owned enterprises, non-state-owned enterprises through mergers and acquisitions to realize the effect of technological innovation is more significant; in terms of the type of M&A transactions, whether domestic or cross-border mergers and acquisitions, large-amount mergers and acquisitions and small-amount mergers and acquisitions can significantly enhance the enterprise's technological innovation.

Finally, the content and conclusions studied in this paper are discussed with the existing studies to elaborate what new things this paper adds to the existing body of knowledge, the strengths and limitations of the study; summarize the conclusions of this study, as well as expanding the policy recommendations on the basis of the core research results.

3. In the second part, it would be beneficial to supplement the literature on corporate mergers and acquisitions.

Response:

Thank you very much for your suggestion. We have added more relevant literature on corporate mergers and acquisitions. The details are at 2.1 Mergers and acquisitions and 2.3 Mergers and acquisitions and technological innovation.

The revised literature related to corporate mergers and acquisitions is as follows:

2.1 Mergers and acquisitions

As a business strategy for enterprises to expand scale, increase market share, optimize resources, and reduce costs, the merger and acquisition effect is a core issue of concern for many parties. The literature on the effects of mergers and acquisitions mainly focuses on merger and acquisition firms and explores the impact of mergers and acquisitions on their market power, productivity and wealth (Wasilewska et al.,2022; Liu et al.,2018; Murray et al., 2017; Pang et al., 2018). Tang et al. (2022) found that mergers and acquisitions can significantly enhance the market power of manufacturing firms from the perspective of digital transformation. Jiang (2021) utilized Thomson Reuters mergers and acquisitions data from 2003 to 2007 and merged it with a database of Chinese industrial enterprises and found that mergers and acquisitions in China not only enhanced the market power of the enterprises but also significantly improved their productivity. By comparing foreign and domestic mergers and acquisitions, Wang et al. (2012) found that foreign mergers and acquisitions can increase the total factor productivity of merger and acquisition firms, but this enhancement is only realized 2-3 years after the merger and acquisition. Using data from the U.S. manufacturing industry, Kim et al. (2019) found that the mergers and acquisition experience of merger and acquisition firms is most positively correlated with productivity. Fich et al. (2018) found that shareholders of merger and acquisition firms gained wealth in merger and acquisition transactions.

2.3 Mergers and acquisitions and technological innovation

In recent years, the relationship between mergers and acquisitions and technological innovation has gradually received attention from academics, and some studies have shown that mergers and acquisitions can drive technological innovation, and merger and acquisition firms gain access to the technological resources of the acquired firms through acquisitions to drive the technological change of the merger and acquisition firms after acquisitions (Li et al.,2022; Chen et al.,2021; Wu et al.,2021; Zhou et al.,2023). Wang et al. (2022) found that firms carry out the redistribution of innovations through mergers and acquisitions and increase their level of innovation (Dushintsky et al.,2010). Ben et al. (2014) argued that innovation often leads to merger and acquisition synergies and that synergies from merger and acquisition activities promote innovation output (Cassiman et al.,2006; Szucs,2014). However, some studies have shown that mergers and acquisitions lead to reduced innovation efficiency (Giudice et al., 2014) and inhibit firms' innovation (Hitt et al.,1991; Blonigen et al.,2000; Federico et al.,2019). Haucap et al. (2019), using data from the European pharmaceutical industry, showed that mergers and acquisitions inhibit firms' R&D investment and reduce the level of firms' technological innovation. Using data from China's manufacturing industry as a sample, Ma et al. (2017) empirically found that nontechnology mergers and acquisitions have a negative impact on the technological innovation of merger and acquisition firms. The empirical results of the impact of mergers and acquisitions on technological innovation are inconsistent, with most studies suggesting that they are influenced by factors such as the motives for mergers and acquisitions and firm and industry heterogeneity. In general, in nontechnology mergers and acquisitions, the acquired firms tend not to provide technical support to the acquiring firms and thus often do not contribute to the technological innovation of the acquiring firms (Ahuja et al.,2001; Cloodt et al.,2006).

4. In part 4, there is a lack of information on indicators and methods for measuring digital transformation.

Response:

Thank you for your suggestion. We have scrutinized the manuscript and added information on measuring digital transformation indicators and methodologies in section 5.4.2 of the revised draft.

The revised measuring digital transformation indicators and methodologies content is below:

The following digital transformation indicators are obtained. First, the annual reports of Chinese A-share listed companies are collected and organized by a Python crawler, and all content was extracted from the data pool used for feature word screening. Second, classical literature, policy documents and research reports with the theme of digital features are referred to, and specific keywords about digital transformation are summarized, mainly including cloud computing technology, big data technology, artificial intelligence technology, blockchain technology and feature words related to the use of digital technology in practice. Third, the word frequency of the feature words is counted to obtain the total word frequency, and the natural logarithm is taken by adding 1 to the total word frequency as a measure of digital transformation.

5. Part introduction should be shortened especially the political one. It is not scientifically sound.

Response:

Thank you very much for your suggestion. We have deleted and revised the political part of the introduction, and revised the introduction according to the logic of real-life scenarios, case examples, research questions and methods. The details are in Part I, introduction.

The contents of the revised introduction are as follows:

In 2010, China has become the largest country in manufacturing industry, but has long been trapped in the plight of serious duplication of industry construction, low industrial concentration, weak independent innovation ability, and weak market competitiveness. Under the pattern of latecomer competition, breaking through the technological blockade, solving the “neck” problem, and realizing subversive innovation is the necessary way for Chinese enterprises to catch up with the technology. However, due to the lack of technical talents and the long technology research and development cycle, it is relatively slow for enterprises to rely on independent innovation to improve their technological level (Sear,2018; Guo et al.,2018). According to the theory of open innovation, enterprises can rapidly acquire heterogeneous knowledge that is difficult to purchase in the factor market through mergers and acquisitions (Li et al.,2019), and utilize external digital resources such as talent, technology and knowledge acquired through mergers and acquisitions to improve the level of technological innovation of enterprises (Cefis et al.,2020). With the growing demand for technological innovation in China, enterprises need to absorb new technological knowledge through cross-industry integration to realize technological innovation faster in order to maintain competitive advantages and grasp new opportunities (Fang et al.,2022), thus a large number of enterprises have emerged to acquire external knowledge through mergers and acquisitions to enhance technological innovation capability.

According to CSMAR data, in terms of quantity and amount, China's merger and acquisition activities have increased over the past decade (Ren et al.,2017). In 2020, the number of China's merger and acquisition transactions reached a record high, with a total of 9,879 merger and acquisition events and a total merger and acquisition value of $409.969 billion, up 168% and 356%, respectively, compared with 2007. However, there are many cases of failure. For example, since 2015, Chengdu Tianxiang Environment Co., Ltd., China's leading environmental protection equipment company, has acquired the U.S. sludge treatment equipment company Centrisys Corporation, Germany's Belfinger Water Treatment (BWT), and Germany's “solid waste giant” Euromonitor Group, but the company went bankrupt and delisted in December 2020 due to a lack of breakthroughs in core technology and poor operations.

Based on the above, this study aims to answer the following questions: Can mergers and acquisitions promote the technological innovation of Chinese enterprises? If so, what is the mechanism? As there are both state-owned and non-state-owned enterprises in China, does the relationship between mergers and acquisitions and technological innovation reflect heterogeneity according to the characteristics of enterprises and types of mergers and acquisitions? To address these questions, this paper identifies and examines the causal effect and influence mechanism between mergers and acquisitions and technological innovation based on the merger and acquisition data of Chinese A-share listed firms in Shanghai and Shenzhen under the framework of the Difference-in-Differences method of empirical testing. This paper further examines the impact of heterogeneity, i.e., firm idiosyncrasies, according to the type of merger and acquisition transactions and the nature of firm ownership.

6. Discussion is rather poor. What is interesting is to discuss your results with already published as well as their importance for the whole scientific community. What is new that you add to the existing body of knowledge, what are strengths and limitations of the study.

Response:

We are very grateful for your valuable suggestions. Our careful reading of the manuscript revealed that there was indeed a lack of discussion of what is new in this study versus the published results and its importance to the scientific community as a whole. Therefore, a discussion has been added to this study in part 6.

The revised discussion is below:

The current research results are quite rich, and this paper builds on this foundation and further enriches the body of knowledge by focusing on Chinese listed companies.

First, at present, academics have conducted many theoretical and empirical studies on the relationship between mergers and acquisitions and technological innovation, but the conclusions are significantly divergent. Taking Chinese A-share listed companies as the research object, this paper finds that corporate mergers and acquisitions have a positive effect on technological innovation, which still holds after a parallel trend test and a placebo test, and further verifies that corporate mergers and acquisitions are the main driving force of technological innovation, which supports the conclusions of the studies by Wu et al. (2022) and Chen et al. (2024). Additionally, this paper finds that the enhancement effect of mergers and acquisitions on technological innovation persists in the long term and becomes increasingly obvious as the postmerger integration process advances. As shown in Figure 2, $\gamma_{6}=0.6477$ is significant at the 1% level, indicating that six years after a merger and acquisition occurs, it can still significantly enhance the technological innovation of the firm, and the economic significance is high.

Second, current scholars focusing on high-tech enterprises and family-owned enterprises believe that mergers and acquisitions can promote technological innovation, which is mainly realized through saving transaction costs, improving enterprise productivity (Chen et al.,2024), reducing R&D risks, and improving R&D efficiency (Ye et al., 2024). This paper verifies that improving productivity is an important path, which supports the current research. Additionally, this paper finds that mergers and acquisitions can also promote technological innovation through two paths, specifically, enriching the level of digital knowledge and enhancing market power, of which the enhanced effect of enterprise digital transformation has the strongest explanatory power, suggesting that it is an important pathway for enhancing technological innovation. This further reveals the mechanism by which mergers and acquisitions promote technological innovation.

Third, current research suggests that firms with high productivity (Liu et al.,2018), strong resource reorganization capabilities (Chen et al.,2023), and few financing constraints (Cheng et al., 2023) are more suitable for mergers and acquisitions. This study contributes by showing that when a merger and acquisition firm is a non-state-owned enterprise, its mergers and acquisitions have a significant positive effect on technological innovation, and the mergers and acquisitions of state-owned enterprises do not have a significant effect on technological innovation. Domestic mergers and acquisitions play a more pronounced role in promoting technological innovation than cross-border mergers and acquisitions, and small-dollar mergers and acquisitions play a more pronounced role in promoting technological innovation than large-dollar mergers and acquisitions. This study further reveals the characteristics of enterprises that are more suitable for promoting technological innovation through mergers and acquisitions from the perspectives of enterprise ownership, merger and acquisition type and merger and acquisition intensity.

Nonetheless, this study has some limitations. First, this study focuses on data from listed companies in China and attempts to consider nonlisted companies in the subsequent period. Second, this study observes the acquiring firms in merger and acquisition transactions, and the traits of the acquired firms, as the other participants in merger and acquisition transactions, may also have an impact on technological innovation. Third, this study is based on data from Chinese listed companies in the Chinese context. There are significant differences in the merger and acquisition behaviors and external conditions of firms in different countries. Therefore, future research can be carried out in the following three ways. First, nonlisted companies can be used as the research object in the subsequent period to comprehensively understand the impact of mergers and acquisitions on technological innovation in Chinese enterprises. Second, the acquired enterprises can be used as the research object to further enrich the body of knowledge on the impact of mergers and acquisitions on technological innovation. Third, to make the results of the study more widely applicable, future enterprise samples should be obtained based on a larger number of countries to conduct empirical tests.

7. The policy recommendations needs to be further improved, and it is suggested that the author further expand the policy based on the core research results.

Response:

Thank you very much for your comments on refining the policy proposal. We have carefully revised and refined the policy recommendations.

The specifics are in the second point of the policy recommendation in Part 7.

The revised policy recommendations are listed below:

Based on the above findings, the following recommendations can be made.

First, the government should provide a strong policy environment and financing support for mergers and acquisitions to provide a source of power for enterprise technological innovation. Banks and other financial institutions should be guided to broaden the financing channels for mergers and acquisitions and provide governmental financing guarantees for enterprises to carry out mergers and acquisitions. Additionally, appropriate policy subsidies and tax exemptions and reductions for mergers and acquisitions should be provided to minimize the risks and costs faced by enterprises in mergers and acquisitions. Enterprises should make full use of the government's supportive policies to support mergers and acquisitions, focus on R&D innovation after mergers and acquisitions, increase R&D investment, cultivate professional and technical talent, and truly transform the technological resources acquired through mergers and acquisitions into the enterprise's own technological strength.

Second, the government should build a digital resource platform; promote the convergence of innovative resources; accelerate the efficient circulation of data, knowledge and other factors of production; strengthen the joint cultivation of schools and enterprises; expand the channels for attracting talent from home and abroad; increase the introduction and training of technical talent; open up the chain of talent from enterprises, universities and scientific research institutes; establish industry-academia-research cooperation teams; gather resources from industry-academia-research; systematically promote the technological research of key areas; give enterprises priority access to new products; and utilize their own influence and social credibility to promote quality products. Enterprises should absorb talent around the core technology after the completion of mergers and acquisitions, promote the process of technology absorption and reinnovation, accelerate the transformation of core technology, emphasize the development of new products, accelerate the promotion of new products, create unique brands belonging to the enterprise, and enhance the competitiveness of the market.

Third, the government should formulate preferential tax and credit policies for mergers and acquisitions of non-state-owned enterprises, provide a liberal merger and acquisition environment, reduce the costs and obstacles of mergers and acquisitions, relax the merger and acquisition approval policy of non-state-owned enterprises, simplify merger and acquisition approval procedures, promote an increase in the merger and acquisition activity of non-state-owned enterprises, and encourage non-state-owned enterprises to initiate or participate in different types of mergers and acquisitions. Enterprises should choose the type of merger and acquisition from the strategic height of technological innovation and the actual situation, seize the opportunity to open overseas markets through cross-border mergers and acquisitions, pay attention to the resource synergy effect through domestic mergers and acquisitions, absorb the high-value resources acquired through large-amount mergers and acquisitions, and accumulate merger and acquisition experience gained through small-amount mergers and acquisitions to lay the foundation for difficult and complicated mergers and acquisitions in the future.

**We have done our best to improve the manuscript, and have made some red-flagged changes in revising the paper that do not affect the content or framework of the paper. We sincerely thank the editor and reviewers for their enthusiastic work and hope that the revisions will be recognized. Thank you again for your comments and suggestions.**
