# Supplementary material for: Research on the impact of enterprise mergers and acquisitions on technological innovation: An empirical analysis based on listed Chinese enterprises
Source: PLoS One. 2024 Nov 27;19(11):e0309569. doi: 10.1371/journal.pone.0309569 (PMC11602071; doi:10.1371/journal.pone.0309569)
Supplement: Attachment — Submitted filename: Response to Reviewers.docx [file pone.0309569.s005.docx]

**Respond to the editor’ comments**

Dear Editor:

Thank you for your letter and the constructive comments on our manuscript, “Research on the impact of enterprise mergers and acquisitions on technological innovation: An empirical analysis based on listed Chinese enterprises” (PONE-D-24-14261R1), in your busy schedule. All of us authors have carefully read the comments that you have given us, and have discussed and revised each of these issues. The following is my list of revisions. In addition, we have resubmitted a new manuscript in the revised state, with the revisions highlighted in red. If there are any incorrect answers or questions in the manuscript, please do not hesitate to let us know.

Journal Requirements:
Please review your reference list to ensure that it is complete and correct. If you have cited papers that have been retracted, please include the rationale for doing so in the manuscript text, or remove these references and replace them with relevant current references. Any changes to the reference list should be mentioned in the rebuttal letter that accompanies your revised manuscript. If you need to cite a retracted article, indicate the article’s retracted status in the References list and also include a citation and full reference for the retraction notice.

Response:

Thank you for your constructive comments. We checked and revised the reference lists of the articles and replaced or deleted the withdrawn papers to ensure that they were complete and correct. References numbered 50 and 65 were replaced with references numbered 20 and 27, respectively.

The main revisions are listed below:

Original reference: 50. Pang J R, Zhou H, Wang W. Determinants and wealth effects of listed companies’ decisions to establish buyout funds. Journal of Financial Research. 2018; 2:153-171.

Replacement of reference: 20. Fich E M, Nguyen T, Officer M. Large wealth creation in mergers and acquisitions. Financial Management. 2018; 47(4):953-991.

Original reference：65. Wang Z C. Which mergers and acquisitions can improve the production efficiency, foreign capital or domestic funded? -Based on experienced evidence of manufacturers' mergers and acquisitions. Contemporary Innovation and Development in Management Science. 2012;53-59.

Replacement of reference: 27. He Y, Chiu Y H, Zhang B. Prevaluating technical efficiency gains from potential mergers and acquisitions in China’s coal industry. Sage Open. 2020;10(3): 2158244020939533.doi: 10.1177/2158244020939533

**We have done our best to improve the manuscript, and have made some red-flagged changes in revising the paper that do not affect the content or framework of the paper. We sincerely thank the editor and reviewers for their enthusiastic work and hope that the revisions will be recognized. Thank you again for your comments and suggestions.**

**Respond to the Reviewer #3’ comments**

Dear reviewer.

Thank you for your decision and constructive comments on my manuscript, “Research on the impact of enterprise mergers and acquisitions on technological innovation: An empirical analysis based on listed Chinese enterprises” (PONE-D-24-14261R1). These comments are valuable for the revision and improvement of our paper, and they are also important guidance for our research. We have carefully considered these comments and revised our thesis for your approval. In the revised original manuscript, all changes are marked in red to facilitate your review.

Revision notes, point-to-point, are given as follows:

1. Suggest incorporating comparative analyses with companies from different countries to provide broader insights into the impact of mergers and acquisitions on technological innovation in diverse contexts.

Response:

Thank you for your suggestion. We combine the U.S., Switzerland and Korea to understand the impact of M&A on technological innovation in different contexts.

The revisions are as follows:

Nonetheless, this study has some limitations. First, this study focuses on data from listed companies in China and attempts to consider nonlisted companies in the subsequent period. Second, this study observes the acquiring firms in merger and acquisition transactions, and the traits of the acquired firms, as with the other participants in merger and acquisition transactions, may also have an impact on technological innovation. Third, the positive impact of M&A on technological innovation in enterprises has been demonstrated in many cases around the world. For example, Qualcomm in the United States successfully acquired Nuvia, a chip architecture design company, and utilized Nuvia's advanced design concepts and technological advantages to build a chip that surpassed the performance of Apple's A17Pro and Snapdragon 8gen3 in 2023; this greatly enhanced the technological innovation level of the enterprise. As an innovation-driven, research and development-based healthcare multinational, Roche Switzerland broadened its molecular diagnostics portfolio through the acquisitions of state-of-the-art U.S.-based bioengineering companies Genentech and GeneMark Diagnostics as a means of driving technological innovation and product development. Through the acquisition of Viv Labs and Harman International Industries, South Korea's Samsung has achieved technological breakthroughs in intelligent voice interaction and autonomous driving, completing the industry chain from software to hardware. However, there are significant differences in M&A behaviors and external conditions in different countries, specifically in terms of economic level, cultural variability, and political influence. Therefore, the impact of M&A on the technological innovation of enterprises is bound to vary among countries. Accordingly, future research can be carried out in the following three ways. First, nonlisted companies can be used as the research object in the subsequent period to comprehensively understand the impact of mergers and acquisitions on technological innovation in Chinese enterprises. Second, the acquired enterprises can be used as the research object to further enrich the body of knowledge on the impact of mergers and acquisitions on technological innovation. Third, in the future, it will be necessary to consider enterprises from countries other than China as research objects and to further compare and analyze the different mechanisms and paths of the influence of M&A on technological innovation among different countries.

2. Consider incorporating case studies or real-world examples to illustrate how successful implementation of these policy recommendations can lead to tangible outcomes in terms of technological innovation and business growth.

Response:

Thank you very much for your suggestion. We include concrete cases and real-world examples of how the implementation of these policy recommendations can lead to tangible results in terms of technological innovation and business growth.

The revisions are as follows:

First, the government should provide a strong policy environment and financing support for mergers and acquisitions to provide a source of power for enterprise technological innovation. Banks and other financial institutions should be guided to broaden the financing channels for mergers and acquisitions and provide governmental financing guarantees for enterprises to carry out mergers and acquisitions. Additionally, appropriate policy subsidies and tax exemptions and reductions for mergers and acquisitions should be provided to minimize the risks and costs faced by enterprises in mergers and acquisitions. Enterprises should make full use of the government's supportive policies to support mergers and acquisitions, focus on R&D innovation after mergers and acquisitions, increase R&D investment, cultivate professional and technical talent, and truly transform the technological resources acquired through mergers and acquisitions into the enterprise's own technological strength. For example, in 2014, the State Council of China issued the Opinions on Further Optimizing the Market Environment for Corporate Mergers and Restructuring, which explicitly encourages financial institutions to carry out mergers and restructuring of financing for businesses; additionally, various types of financial investment entities can participate in mergers and restructuring by setting up equity investment funds, venture capital funds, industrial investment funds, merger and acquisition funds, etc. The use of diversified fundraising channels can help reduce the financial constraints on merging and acquiring enterprises, safeguard further R&D investment by enterprises, and thus promote their innovative activities. According to CSMAR database statistics, in 2014, the number of M&A cases announced by A-share listed Chinese enterprises in Shanghai and Shenzhen was 5,689, and the number of patent applications by enterprises was 16,955. After the opinion was put forward, the number of M&A cases reached 7,945 in 2015, and the number of patent applications was 26,766, showing growth rates of 40% and 58%, respectively.

Second, the government should build a digital resource platform; promote the convergence of innovative resources; accelerate the efficient circulation of data, knowledge and other factors of production; strengthen the joint cultivation of schools and enterprises; expand the channels for attracting talent from home and abroad; increase the introduction and training of technical talent; open up the chain of talent from enterprises, universities and scientific research institutes; establish industry-academia-research cooperation teams; gather resources from industry-academia-research; systematically promote the technological research of key areas; give enterprises priority access to new products; and utilize their own influence and social credibility to promote quality products. Enterprises should absorb talent around the core technology after the completion of mergers and acquisitions, promote the process of technology absorption and reinnovation, accelerate the transformation of core technology, emphasize the development of new products, accelerate the promotion of new products, create unique brands belonging to the enterprise, and enhance the competitiveness of the market. For example, Shanxi Province in China, with its rich resources of universities and research institutes, has been steadily moving forward in recent years in terms of the intensity of R&D investment in general and by universities, the number of geographic contracts for technology output in the technology market, the amount of cooperation between enterprises and universities and research institutes, and the number of papers jointly published by enterprises and universities and research institutes. These efforts have laid a solid foundation of science and technology and industrial innovation for the cultivation and development of high-quality products. Regarding enterprises, Tencent successively acquired Riot Games, Epic Games and Supercell and then quickly absorbed resources and launched game products such as League of Legends, Clash of Clans, and Clash Royale; it thereby smoothly entered the game industry and seized the market position, and it has now become the world's largest game publisher.

Third, the government should increase the promotion of technological mergers and acquisitions by enterprises, focus on the selection of targets for mergers and acquisitions by enterprises, and encourage enterprises to achieve technological innovation through technological mergers and acquisitions. It should implement merger and acquisition encouragement policies in a prudent manner, avoid concentrating too many merger and acquisition resources on large enterprises and state-owned enterprises, and provide a level playing field for the industry; in particular, it should guide small and non-state-owned enterprises to cultivate their own core competencies, which are symbolized by their technologies and patents. Enterprises should select M&A objects by strategically considering technological innovation and the actual situation. In particular, enterprises with technology as the main driver of profit should give full play to their absolute advantages and should pursue merges and acquisitions of other technology enterprises to combine strengths; after completing a merger or acquisition, the company should immediately begin the integration and utilization of innovative resources, accelerate the process of technological innovation, and ultimately achieve innovation in key technological fields. For example, Huawei Technologies Co., Ltd. acquired Caliopa, a silicon photonics technology developer in the data communication and telecom markets, and then quickly absorbed the core technology and innovation resources of the target enterprise and leveraged new-generation information technologies such as big data, the Internet of Things, and blockchain to enhance its independent innovation capability, make breakthroughs in core technologies, and solve bottleneck problems. Finally, Huawei successfully researched and developed its own 5G base station core chip in September 2023, released the Kirin 9000S processor, achieved the localization of the 5G chip, and thereby occupied a leading position in global 5G technology.

**We have done our best to improve the manuscript, and have made some red-flagged changes in revising the paper that do not affect the content or framework of the paper. We sincerely thank the editor and reviewers for their enthusiastic work and hope that the revisions will be recognized. Thank you again for your comments and suggestions.**

**Respond to the Reviewer #4’ comments**

Dear reviewer.

We would like to thank you very much for your professional comments on our manuscript "Research on the impact of enterprise mergers and acquisitions on technological innovation: An empirical analysis based on listed Chinese enterprises" (PONE-D-24-14261R1). These comments are valuable for the revision and improvement of our paper, and they are also important guidance for our research. Based on your suggestions, we have made extensive corrections to our previous manuscript, with corrections marked in red for your review.

The following is a point-by-point response to your comments.

I think the paper is interesting, the empirical methodology and results are sound and overall the paper in it's current form is good. I have one comment concerning the last policy recommendation. Your paper does show gains from M&A and firm consolidations, however there are known welfare costs to mergers and market monopolization. So I would be careful in recommending a blanket easing of mergers. I suggest you reformulate this last recommendation, to reflect something along the lines that the policy makers should internalize the technological innovation aspect (documented in your paper) in decision making concerning mergers and acquisition.

Response:

We are very grateful for your valuable comments. Regarding the last policy recommendation, we have revised it taking into account your comments, and the specific revisions are as follows.

Third, the government should increase the promotion of technological mergers and acquisitions by enterprises, focus on the selection of targets for mergers and acquisitions by enterprises, and encourage enterprises to achieve technological innovation through technological mergers and acquisitions. It should implement merger and acquisition encouragement policies in a prudent manner, avoid concentrating too many merger and acquisition resources on large enterprises and state-owned enterprises, and provide a level playing field for the industry; in particular, it should guide small and non-state-owned enterprises to cultivate their own core competencies, which are symbolized by their technologies and patents. Enterprises should select M&A objects by strategically considering technological innovation and the actual situation. In particular, enterprises with technology as the main driver of profit should give full play to their absolute advantages and should pursue merges and acquisitions of other technology enterprises to combine strengths; after completing a merger or acquisition, the company should immediately begin the integration and utilization of innovative resources, accelerate the process of technological innovation, and ultimately achieve innovation in key technological fields.

**We have done our best to improve the manuscript, and have made some red-flagged changes in revising the paper that do not affect the content or framework of the paper. We sincerely thank the editor and reviewers for their enthusiastic work and hope that the revisions will be recognized. Thank you again for your comments and suggestions.**
